# Supplementary material for: Effect of Mosses and Long-Term N Addition on δ13C and δ18O Values of Respired CO2 Under a Temperate Forest Floor
Source: Plants (Basel). 2025 Aug 31;14(17):2707. doi: 10.3390/plants14172707 (PMC12430311; doi:10.3390/plants14172707)
Supplement: Supplementary file 1 [file plants-14-02707-s001.zip › plants-3776068-Supplementary Materials.pdf]

**Supplementary Materials:**

# Effect of mosses and long-term N addition on $\delta^{13}C$ and $\delta^{18}O$ values of respired $CO_2$ under a temperate forest floor

Xingkai Xu[1,2*], Yuhua Kong[3], Erpeng Feng[1,3], Jin Yue[1], Weiguo Cheng[4], Dmitriy Khoroshaev[5] and Sergey Kivalov[5]

1 State Key Laboratory of Atmospheric Environment and Extreme Meteorology, Institute of Atmospheric Physics, Chinese Academy of Sciences, Beijing 100029, China yuejin@mail.iap.ac.cn (J.Y.); 15236902569@163.com (E.F.)
2 Department of Atmospheric Chemistry and Environmental Science, College of Earth and Planetary Sciences, University of Chinese Academy of Sciences, Beijing 100049, China
3 College of Forestry, Henan Agricultural University, Zhengzhou 450046, China; y.kong@henau.edu.cn (Y.K.);
4 Faculty of Agriculture, Yamagata University, Tsuruoka 997-8555, Japan; cheng@tds1.tr.yamagata-u.ac.jp (W.C.)
5 Institute of Physicochemical and Biological Problems in Soil Science, Russian Academy of Sciences, Pushchino 142290, Russia; d.khoroshaev@pbcras.ru (D.K.), kivalov@pbcras.ru (S.K.)

* Correspondence: xingkai_xu@mail.iap.ac.cn (X.X.).

**Table S1.** Changes in the $\delta^{13}C$ and $\delta^{18}O$ values of respired $CO_2$ as well as $CO_2$ and $CH_4$ fluxes prior to and after the removal of moss blankets[a)].

| | $CO_2$ flux (μmol $m^{-2}$ $s^{-1}$) | $CH_4$ flux (nmol $m^{-2}$ $s^{-1}$) | $\delta^{13}C$ values of respired $CO_2$ (‰) | $\delta^{18}O$ values of respired $CO_2$ (‰) |
|---|---|---|---|---|
| Prior to the removal of moss blankets | 0.449±0.106a | −(0.605±0.334)a | −(27.93±1.08)a | −(22.13±5.98)a |
| After the removal of moss blankets | 0.373±0.110a | −(0.526±0.318)b | −(28.13±3.28)a | −(28.93±8.68)a |

[a)] Means ± standard errors ($n$ = 4). Within each column, means followed by different letters were significantly different at the $P$ < 0.05 level (two rails).

**Table S2.** Means and standard errors (in bracket) of δ13C values of $CO_2$ respired from N-treated and non-treated plots with and without mosses and the results of ANOVA analysis.

| Treatments | 2024 | 2021 | 2019 |
|---|---|---|---|
| With mosses | | | |
| Control | −27.76(0.59) | −22.21(0.52) | −21.86(0.13) |
| Low N | −27.26(0.91) | −24.83(0.45) | −22.21(0.44) |
| High N | −26.67(0.83) | −23.58(0.32) | −21.39(0.24) |
| Without mosses | | | |
| Control | −27.05(0.52) | −21.26(0.51) | −20.47(0.25) |
| Low N | −26.28(0.71) | −23.38(0.31) | −21.44(0.78) |
| High N | −26.30(0.87) | −22.47(0.37) | −21.08(0.27) |
| ANOVA with moss and treatment as fixed factors (*P* value) | | | |
| Moss | 0.278 | 0.003 | 0.025 |
| Treatment | 0.471 | 0.000 | 0.236 |
| Treatment × Moss | 0.920 | 0.841 | 0.434 |

**Table S3.** Means and standard errors (in bracket) of $\delta^{18}O$ values of $CO_2$ respired from N-treated and non-treated plots with and without mosses and the results of ANOVA analysis.

| Treatments | 2024 | 2021 | 2019 |
|---|---|---|---|
| With mosses | | | |
| Control | −23.66(4.00) | −12.86(1.52) | −5.16(1.70) |
| Low N | −10.45(1.63) | −14.95(2.13) | −12.08(2.42) |
| High N | −20.26(0.77) | −15.80(1.76) | −14.27(2.56) |
| Without mosses | | | |
| Control | −26.32(2.97) | −13.45(0.91) | −6.35(1.51) |
| Low N | −14.91(4.37) | −17.46(2.23) | −10.42(3.17) |
| High N | −17.04(4.24) | −16.32(1.06) | −19.07(1.08) |
| ANOVA with moss and treatment as fixed factors (*P* value) | | | |
| Moss | 0.635 | 0.389 | 0.430 |
| Treatment | 0.006 | 0.150 | 0.000 |
| Treatment × Moss | 0.489 | 0.801 | 0.356 |
